# Ecological Engineering for the Optimisation of the Land-Based Marine Aquaculture of Coastal Shellfish

**DOI:** 10.3390/ijerph17197224

**Published:** 2020-10-02

**Authors:** Catharina J. M. Philippart, Kiki E. M. Dethmers, Johan van der Molen, André Seinen

**Affiliations:** 1 Department of Coastal Systems, Royal Netherlands Institute for Sea Research and Utrecht University, P.O. Box 59, 1790 AB Den Burg (Texel), The Netherlands; kiki.dethmers@nioz.nl (K.E.M.D.); johan.van.der.molen@nioz.nl (J.v.d.M.); 2 Meromar Seafoods B.V., Celsiusstraat 15, 8861 NE Harlingen, The Netherlands; andre@meromar.nl

**Keywords:** *Cerastoderma edule*, survival, growth, phytoplankton, microphytobenthos, median grain size, silt content, flushing rates

## Abstract

Whilst the demand for nutritious and sustainable seafood is increasing, fishing yields are declining due to overfishing and climate change. The inshore aquaculture of marine molluscs—e.g., the suspension-feeding cockle *Cerastoderma edule* for NW Europe—might be an alternative practice if cost-effective and nature-based technology enhances growth and survival. Our inshore experiments revealed that increasing the seawater residence time resulted in improved water quality. The reduction in sediment loads and stimulation of pelagic microalgal growth resulted in enhanced shell growth and meat content of the wild cockles seeded into the system. Shorter residence times resulted also in an increase in benthic microalgae, but the concurrent increase in silt content of the sediment appeared to hamper effective filtration by cockles. The growth conditions (with respect to the water and sediment quality) for the inshore cultivation of cockles can indeed be improved by means of ecological engineering, suggesting that the inshore aquaculture of marine shellfish can provide sustainable food and income for coastal communities, in particular when the shellfish farms are located in low-lying salinized coastal areas where common agriculture practices are no longer profitable. The involvement of the shellfishery industry was and will be crucial for studying and understanding the complex practice of cockle cultivation.

## 1. Introduction

Providing healthy and nutritious food, in particular animal proteins, for a growing human population living in coastal areas is one of the main challenges the world is facing [1]. The sustained sea-level rise in a warming world has led to serious salinization in coastal lowlands, jeopardizing common agriculture practices [2]. In addition, marine fishing yields have declined due to overfishing and the impacts of climate change [3,4], contributing to a reduction in seafood consumption as a source of high-quality nutrients. These developments call for innovative and sustainable means of aquaculture production to secure a stable long-term food supply of seafood with minimum ecological impacts [5,6].

The inshore aquaculture of marine molluscs might be an alternative practice to sustainably exploit low-lying salinized coastal areas and contribute to the demand for seafood at the same time. When introducing a new product, it is important that both its environmental safety as well as commercial yield are recognized. For example, targeting a species of local origin prevents the spreading of invasive species [7]. The target species should occur in high densities, be fast-growing, be low in terms of maintenance effort, and be easy to market [8]. Furthermore, the farm should make use of natural forces (such as a natural supply of food and tides to flush the system) to keep the ecological and carbon footprint as well as the exploration costs as low as possible [9]. Last but not least, the target species should be selected on the basis of its capacity to grow under warming conditions [10].

The edible cockle *Cerastoderma edule*, an ecologically and economically important bivalve species found along the north-eastern Atlantic coast, is a possible candidate for sustainable inshore culturing in north-western Europe. It is one of the most abundant mollusc species, with recorded densities of more than 10,000 per m^2^ in some bays and estuaries [11]. This species has a 1- to 2-year generation time [12] and can potentially grow to a commercial size (shell > 25 mm; [13]) within 1 year [14]. Cockles (*Cerastoderma edule*) provide meat and shell by-products, with a potential value of €11.3 M per year [15]. With Senegal in western Africa as the southernmost limit of its geographical distribution area [16] and having a thermal range of 4° to 38° in NW Europe [17], this species is likely able to withstand the impacts of warming in more northern areas.

Cockles are suspension-feeding bivalves that obtain food particles from the water–sediment interface. During the filtration of suspended particulate matter (SPM), cockles reject inorganic material and ingest organic material, with living microalgae being their most important food source [18]. This selection mechanism allows their food intake to remain constant at increasing SPM concentrations, until the concentration becomes so high that the selection is no longer effective [19]. Cockles also feed on microphytobenthos [20,21], which occurs mainly on tidal flats and thrives in shallow and clear waters [22]. If living in the proximity of river outflows, cockles also take advantage of the local supply of freshwater algae [21].

Cost-effective and nature-based technology may improve the growth of cockles and subsequently the yield of cockle farms. Increasing the residence time of seawater in weak currents before supplying it to the cockles, for example, can reduce the silt content of coastal waters and, consequently, improve the food uptake efficiency of the cockles. Pelagic microalgal biomass can increase up to 67% per day under optimal light and nutrient conditions [23]. Such an increase in food density could be artificially enhanced by increasing the water residence time in the cultivation system. A decrease in the turbidity of the water can also stimulate the benthic food supply for the cockles when the growth of benthic microalgae is light-limited. This could result in a faster shell growth (mm per day), higher meat content (µg AshFreeDryWeight (AFDW) per mm^3^ shell volume), and higher production capacity (number of cockles per m^2^ per year; m^2^ of cockle farmland per m^3^ of water).

Here, we present the results of experiments where we examined the impact of flushing rate on the environmental (growth) conditions and cockle performance indices at an inshore testing site. This site was built in a former polder on Texel, the westernmost island in the Wadden Sea, which is a coastal sea adjacent to the North Sea. Seawater was let into the site by means of siphoning and transported through a series of basins (raceways) by means of a system of lock gates and valves. The locks and valves were used to control the flow through the raceways. This study addressed the following questions: (i) Do flushing rates affect the amount of pelagic and benthic microalgae as a food source for cockles? (ii) Do flushing rates affect the net sedimentation of the suspended silt fraction of the water? (iii) If flushing rates influence the growth conditions, do they also modify cockle survival, shell growth, and meat content?

## 2. Material and Methods

### 2.1. Study Site and Experimental Set-Up

**Figure 1:**
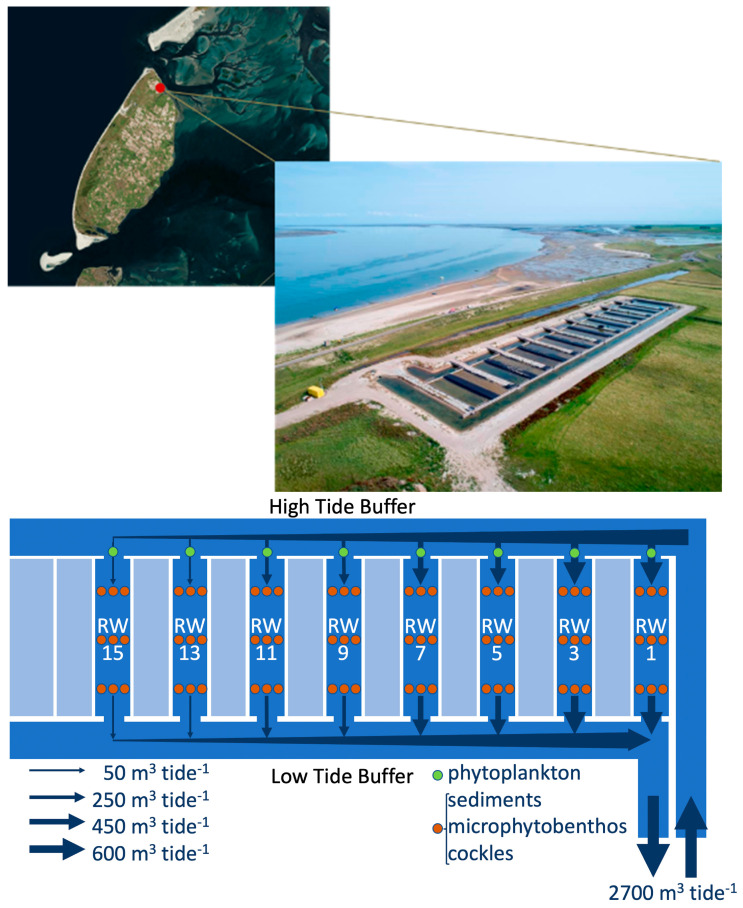
Location of the site; outline of the experimental set-up; and position of the sampling points for phytoplankton (green dots) and for the sediment, cockles, and microphytobenthos (red dots) within the raceways (RW).

The experiments were carried out in a hydrodynamic field laboratory constructed in the “Polder Wassenaar” area on Texel (Figure 1). The experiments were performed in 8 (numbers 1, 3, 5, 7, 9, 11, 13, and 15) out of the 16 raceways of the aquaculture system, with each raceway being 30 m long, 5 m wide, and 0.48 m deep (below the Dutch Ordinance Level). With the incoming tide, the seawater flowed into the so-called high-tide buffer (HTB) via an underground supply (siphon) through the sea dyke and subsequently into the raceways via small sluices. With the outgoing tide, the water flowed back into the Wadden Sea from the raceways via small sluices into the low-tide buffer (LTB) and then via the siphon. Valves within the siphon ensured that the water was always flowing in the same direction. The raceways that were not used for the experiment (numbers 2, 4, 6, 8, 10, 12, 14, and 16) were permanently closed at both ends.

The flushing rate (m^3^ per tide) in each raceway was projected by means of the hydrodynamic SOBEK modelling software (Deltares, Delft, The Netherlands; version 3.7.13), which was originally developed for predicting and controlling irrigation systems, river and channel flows, water levels, and the surface water quality [24,25]. Based on the dimensions of the aquaculture system and the tidal amplitudes in the adjacent Wadden Sea, this model calculated the amount of inflowing water by adjusting the inflow and outflow opening of the sluices at both ends of each raceway. The model predictions were validated with data on tidal heights within the system. Flushing rates were set on 600, 450, 250, and 50 m^3^ water per tide, with two replicates (raceways) for each flushing rate situated next to each other. The opening of the sluices in the system was set and fixed from 6 November 2018 until the end of the experiments.

**Figure 2:**
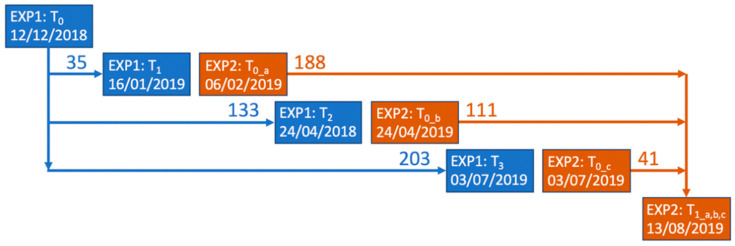
Sampling scheme for cockles divided into two experiments. For Experiment 1, the cockles were all transplanted into the enclosures on 12 December 2018 (in 9 enclosures per raceway, 72 enclosures in total), and sampled on 16 January, 24 April, and 3 July 2019 (from 3 enclosures per raceway, with 24 enclosures per sampling period). For Experiment 2, the cockles were transplanted on 6 February, 24 April, and 3 July 2019 (in 3 enclosures per raceway, with 24 enclosures per sampling period) and all were sampled on 13 August 2019 (72 enclosures in total).

Each raceway contained 9 circular enclosures for cockles with an inner diameter of 48.2 cm, consisting of a 7 cm-high PVC pipe with a 15 cm-high gauze (6.3 × 6.3 mm mesh) attached to it to contain the cockles. These enclosures were pushed into the sediment to a depth of 7 cm, leaving 8 cm of gauze above the sediment surface. Two parallel experiments were run, where enclosures being sampled for one experiment were filled with fresh cockles for the other experiment. “Experiment 1” had a common seeding date (12 December 2018) and three sampling dates (16 January, 24 April, and 3 July 2019), while “Experiment 2” had three seeding dates (6 February, 24 April, and 3 July 2019) and one common sampling date (13 August 2019) (Figure 2).

For both experiments, each enclosure was seeded with 40 juvenile cockles from two separate wild stocks, which were originally obtained from nearby tidal flats, collected by local cockle fishermen in November 2018 (used for transplants on 12 December 2018 and 6 February 2019) and April 2019 (used for transplants on 24 April and 3 July 2019). Cockles that were found dead within the enclosures in Experiment 1 (on average, 1 or 2 per enclosure) were removed and replaced by living ones from the stock on 14 December 2018. The cockles and environmental conditions were regularly sampled throughout the experimental period (from 12 December 2018 to 13 August 2019, Supplementary Table S1).

The siphon was cleaned regularly (once every 2 to 3 weeks), removing fouling to ensure a constant water flow throughout the experimental period. The inflow of seawater was halted approximately once a week for various reasons, including the maintenance of the siphon, maintenance of the raceways, during extreme high tides (to prevent the system from overflowing), and during very low temperatures (to prevent the cockles from freezing when emerged). The interruptions lasted from a few hours to a maximum of 2 days per event. The sluices were closed during the dredging of the high-tide buffer (to remove the surplus of sediment), which prevented turbid waters from entering the raceways.

### 2.2. Sampling Methodology

To determine the phytoplankton concentrations, water samples were taken from the high-tide buffer in front of each of the sluices connecting the HTB with the individual raceways at the time when water started to enter raceway 15 (Supplementary Table S1). The water samples were kept cool and dark until filtration, generally within 4 h of sampling. A subsample of 500 mL from each sample was filtered over a pre-heated Whatman^®^ GF/F filter (with a mesh size of 0.7 µm). The material remaining in the filter was then dissolved in 20 mL of acetone (90%), after which the concentration of chlorophyll-a in this solution was measured with a F-2500 Fluorescence Spectrophotometer (Hitachi High-Technologies Corporation, Wokingham, United Kingdom). The measured concentration was then converted to the concentration in the water (mg CHLa m^−3^).

The microphytobenthos concentrations and sediment characteristics were determined from sediment samples taken in the enclosures in January, April, and July 2019 (Supplementary Table S1). The top 4 cm of the sediment was sampled three times with a sampling tube (17 mm diameter) in each enclosure and jointly stored in a glass jar. Upon arrival at the laboratory, the samples were immediately lyophilized and stored in the dark until further analyses could be performed.

The microphytobenthos concentrations in the sediment samples were determined by adding 20 mL of acetone (90%) to 2 g of the lyophilized sediment. After centrifugation (separating solids and liquids), the liquid (acetone containing the dissolved chlorophyll-a) was diluted the following day and the concentration of chlorophyll-a was measured in 0.02 mL of this dilution (in μg chlorophyll-a per mL liquid), using a F-2500 Fluorescence Spectrophotometer (Hitachi High-Technologies Corporation, Wokingham, United Kingdom). These measured concentrations were converted to the concentrations in the sediment (μg CHLa g^−1^).

A weighed portion of homogenized lyophilized sediment was put through a 2 mm sieve (produced in-house by NIOZ) and placed in 15 mL polypropylene auto-sampler tubes (Beckman Coulter, Indianapolis, United States) for the determination of the other sediments’ particle size composition. Purified water (RO water, purified by reverse osmosis) was added and the sample was shaken vigorously on a vortex mixer for 30 s. The median particle size (μm) and proportion of silt (fraction of particles smaller than 63 μm) of the sediments were determined using a LS 13 320 particle size analyzer and autosampler (Beckman Coulter, Indianapolis, United States). This device measures particle sizes in the range of 0.04–2000 μm in 126 size classes, using laser diffraction (780 nm) and polarization intensity differential scattering (PIDS™) (450, 600, and 900 nm) technology.

The cockle performance indices (total number, average shell length, average shell volume, average meat content, and total biomass) were determined from the surviving individuals (Supplementary Table S1). All the remnants of dead cockles were counted and removed. Survival was calculated as the percentage of live cockles remaining (from *n* = 40 seeded cockles) in each sampled enclosure. The volume of the shell (SV; mm^3^) was calculated as pi/6 (length × width × height) [26]. The meat was removed from a subset of individuals from each enclosure (all living cockles except 3 per enclosure batch), and the ash-free dry weight (mg AFDW) of the meat was determined as the difference between the dry weight (mg DW; 48 h at 60 °C) and the ash weight (mg AW; 5 h at 560 °C). The cockle meat content (mg AFDW mm^−3^) was calculated as the ash-free dry weight of the meat (mg AFDW) divided by the shell volume (mm^3^). The total biomass per enclosure (mg AFDW) was calculated as the total shell volume (mm^3^ per enclosure) times the average cockle meat content (mg AFDW mm^−3^). EU regulations apply to live non-human vertebrate animals and live cephalopods, which implies that no ethical approval of our institution’s animal care and usage committee was necessary for our experiments described in this article.

### 2.3. Statistical Treatment and Analysis

First, we tested whether the observations of the various environmental conditions and cockle performance indices could be best explained by hypotheses stating that:

**H1.**  There is one similar seasonal pattern for all raceways;

**H2.**  TThere is one seasonal pattern, with an additional effect of raceway;

**H3.**  TThere is one seasonal pattern, with an additional effect of flushing rate;

**H4.**  TThere is one seasonal pattern, with the additional effects of flushing rate and relative distance to inlet;

**H5.**  TThere are different seasonal patterns per raceway;

**H6.**  TThere are different seasonal patterns per flushing rate;

**H7.**  TThere are different seasonal patterns per flushing rate, with an additional effect of relative distance to inlet.

To test these hypotheses, we modelled the data by means of Generalized Additive Mixed Effects Modelling (GAMM). To compare the model fits, we followed an Information Theoretic approach [27] and calculated the differences Δ*i* between the Akaike Information Criterion (AIC) of each model and the minimum AIC. [27] state that the level of empirical support for model *i* is substantial if Δ*i* is between 0 and 2 (these are models with similar AICs to the optimal model), considerably less if Δ*i* is between 4 and 7, and essentially none if Δ*i* is larger than 10.

Testing the latter three hypotheses (H5–H7), including an interaction term between smoothers, was possible only with large enough data sets, such as that of phytoplankton (Table 1). To enable a full comparison, the data were analyzed with all the effects both as a factor and as a smoother. The analyses of the other variables were restricted to testing the effects (raceway, flushing rate, position in the HTB) as factors only.

We also calculated the Akaike weights *w_i_* [26], which have the convenient ability that they can be interpreted as the probability that a given model is judged the best model upon repeated sampling. If the weight for a particular model has a value of 0.75, for example, this implies that this model has a probability of 75% of being the best model within the series of models tested.

Second, we compared the environmental conditions with the cockle performance indices by analyzing the correlations between the values of the coefficients for the distance to inlet, as derived from the best GAMM model, assuming that the seasonal patterns and distance to inlet are additive factorial effects (H2). The outcomes of this correlation matrix are summarized by means of principal component analyses (PCA), where the 1st principal component (PC1) is the direction along which the samples show the largest variation and the 2nd principal component (PC2) is the direction uncorrelated to the first component along which the samples show the largest variation [28].

Statistical analysis was performed using R (version 3.6.2; [29]) in an R Studio environment (version 1.2.5033; [30]), using the mgcv package (version 1.8–31; [31]) for generalized additive mixed modelling and the MuMIn package (version 1.43.17; [32]) for calculating the Akaike weights.

## 3. Results

### 3.1. Phytoplankton

**Figure 3:**
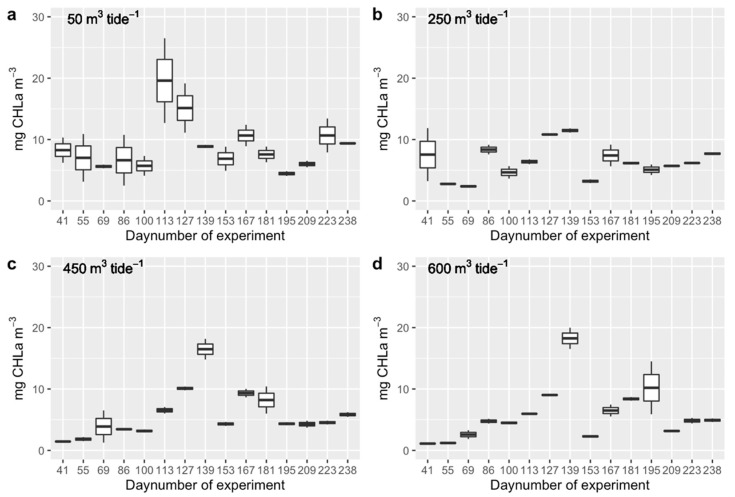
Phytoplankton concentrations (mg CHLa m^−3^) at the flushing rates of (**a**) 50 m^3^ per tide, (**b**) 250 m^3^ per tide, (**c**) 450 m^3^ per tide, and (**d**) 600 m^3^ per tide at sampling periods from 29 January 2019 (day number of experiment = 44) to 5 August 2019 (day number of experiment = 238). See Figure 1 for the locations of sampling points and Table S1 for the number of samples per sampling period.

The average phytoplankton biomass in the high-tide buffer (HTB) increased from 3.20 ± 3.20 mg CHLa m^−3^ in early February 2019 to 13.77 ± 4.23 mg CHLa m^−3^ during the spring bloom at the end of April 2019 (Figure 3). The pelagic microalgal biomass subsequently decreased to 4.17 ± 2.13 mg CHLa m^−3^ in mid-May, followed by an increase to 8.47 ± 2.28 mg CHLa m^−3^ at the end of May 2019, and was variable from then onwards until 6.96 ± 1.87 mg CHLa m^−3^ at the end of the sampling period in early August 2019.

The variation in the phytoplankton biomass during the experiment along the HTB was best described by means of a model that took the interaction between seasonal dynamics and raceway number into account (Table 2). Apparently, the spring bloom was enhanced (occurring earlier in the year) in the high-tide buffer following the direction of the main current (Figure 3; Supplementary Table S2a). These findings imply that the cockles that were transplanted in the downstream raceways profited earlier from the pelagic food supply than the cockles in the upstream raceways.

### 3.2. Microphytobentos

**Figure 4:**
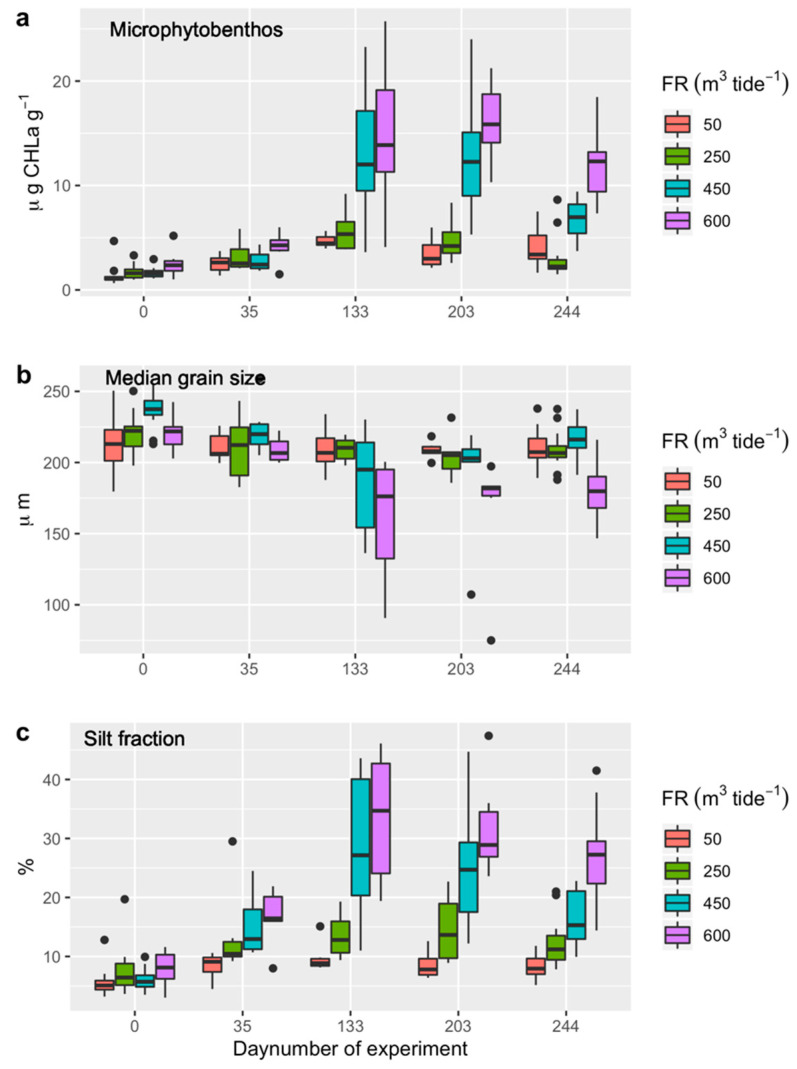
Environmental conditions at various flushing rates (FR is 50, 250, 450, or 600 m^3^ per tide) at sampling periods during the experimental period from 12 December 2018 (day number of experiment = 0) to 13 August 2019 (day number of experiment = 244). (**a**) Microphytobenthos concentrations (µg CHLa g^−1^), (**b**) median grain size (µm), and (**c**) silt fraction (%) of the sediment sampled in the enclosures in the raceways. See Figure 1 for the locations of sampling points and Supplementary Table S1 for the number of samples per sampling period.

The biomass of microphytobenthos, averaged over all raceways, generally increased from 1.75 ± 0.84 mg CHLa g^−1^ in mid-December 2018 to 9.57 ± 6.67 mg CHLa g^−1^ by the end of April 2019 (Figure 4a). The benthic microalgal biomass was similarly high (9.33 ± 6.62 mg CHLa g^−1^) in early July 2019, but decreased to 6.45 ± 4.24 mg CHLa g^−1^ by mid-August 2019.

The values of the Akaike weights *w_i_* indicated that model M3 (the microphytobenthic biomass variation was due to the added effects of seasonality and flushing rate) had the highest probability (65%) of being the best model tested, with substantial support for model M4 (there was an additional effect of the relative position of the raceway towards the inlet of seawater) (31%) as well (Table 3). The results suggest that the biomass of microphytobenthos increased with increasing the flushing rate, and that the upstream raceways have a higher benthic microalgal biomass than the downstream ones with a similar flushing rate (Figure 4a; Supplementary Table S2b).

### 3.3. Sediment Characteristics

The median grain size (MGS) of the sediment, averaged over all the raceways, decreased from 223 ± 15 µm in mid-December 2018 to 191 ± 36 µm at the end of April 2019, followed by an increase from 192 ± 34 µm in early July 2019 to 204 ± 19 µm in mid-August 2019 (Figure 4b). The variation in MGS was best explained by model M3 (with a probability of 71% of being the best model), with substantial support for model M4 (with a probability of 24%) as well (Table 3). Raceways with a flushing rate of 600 m^3^ per tide had relatively low MGS values compared to the other flushing rates (Figure 4b; Supplementary Table S2c).

The average silt fraction (SF) of the sediment increased from 6.7 ± 2.7% in mid-December 2018 to 21.3 ± 13.4% at the end of April 2019, followed by a decrease from 20.1 ± 11.8% in early July 2019 to 16.0 ± 8.5% in mid-August 2019 (Figure 4c). The variation in SF was best explained by model H4 (with a probability of 70% of being the best model; Table 3). The SF appeared to increase with the increasing flushing rates, and it was higher for upstream raceways then for downstream raceways with similar flushing rates (Figure 4c; Supplementary Table S2d).

### 3.4. Cockles

#### 3.4.1. Juvenile Stock

**Figure 5:**
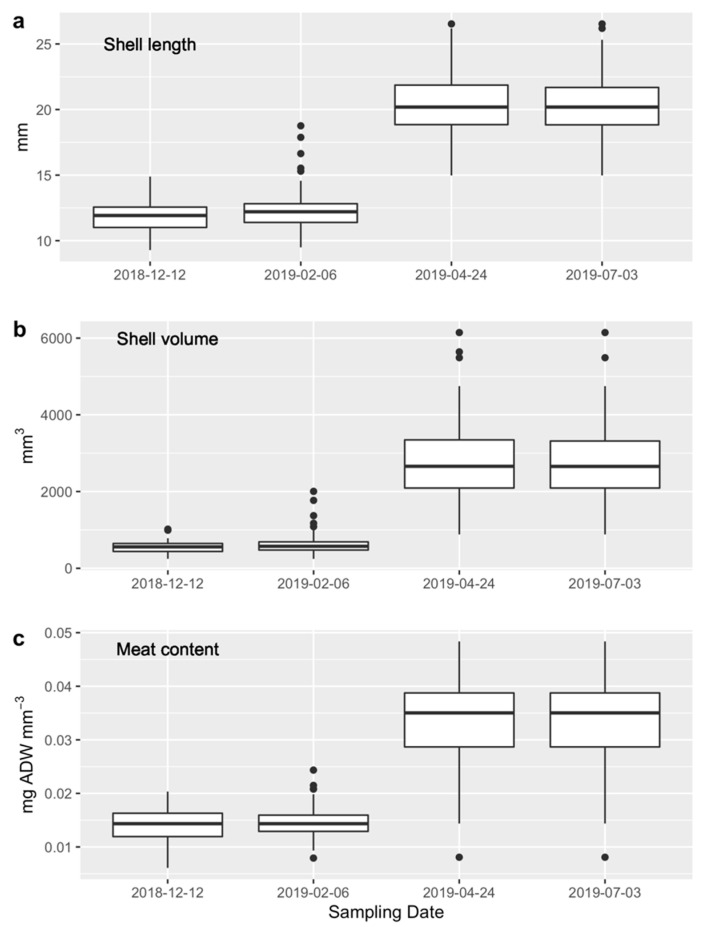
(**a**) Shell length (mm), (**b**) shell volume (mm^3^), and (**c**) meat content (mg AFDW mm^−3^) of a subsample of cockles taken from the stock used for the transplants on 12 December 2018 (common t_0_ for Experiment 1) and on 2 February 2019, 24 April 2019m and 3 July 2019 (t_0_ for Experiment 2, respectively). The number of subsampled cockles for shell allometry ranged between 36 (December 2018) and 120 (February 2019) individuals, and for cockle condition between 27 and 111 individuals at the same dates.

The shell length (mm), shell volume (mm^3^), and meat content (mg AFDW mm^−3^) of the first juvenile stock used for the transplants were lower (ca. 12 mm, ca. 600 mm^3^, and ca. 0.014 mg AFDW per mm^3^) than those of the second stock (ca. 20 mm, ca. 2750 mm^3^, and ca. 0.033 mg AFDW per mm^3^) (Figure 5).

#### 3.4.2. Experiment 1

**Figure 6:**
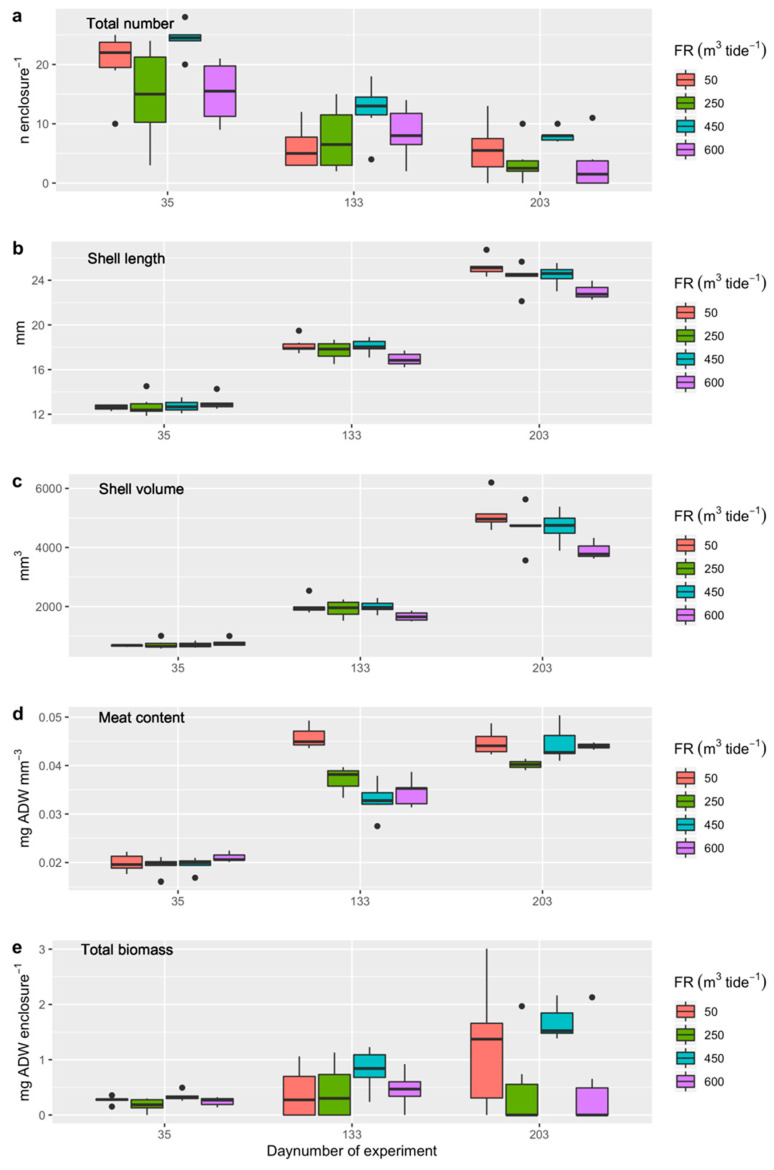
(**a**) Cockle density (numbers per enclosure), (**b**) shell length (mm), (**c**) shell volume (mm^3^), (**d**) meat content (mg AFDW mm^−3^), and (**e**) total cockle biomass (mg ADW per enclosure) for Experiment 1, where all the cockles were transplanted into the enclosures on 12 December 2018 (in 9 enclosures per raceway, 72 enclosures in total) and sampled after 35, 133, and 203 days.

The number of cockles in the enclosures decreased over time from 12 December 2018 onwards, with a survival of 47 ± 17% for cockles sampled in January 2019, 21 ± 12% for cockles sampled in April, and 13 ± 10% for cockles sampled in July 2019 (Figure 6a). The variation in density (number in enclosures) was best explained by model M2 (having the lowest AIC and a probability of 39% of being the best model) (Table 4). The survival in the raceways with a flushing rate of 450 m^3^ per tide was significantly higher (>4%) compared to the survival in the raceways with other flushing rates (Figure 6a; Supplementary Table S3a).

The shell length of the cockles generally increased over time from 12.78 ± 0.62 mm on 16 January 2019 to 24.38 ± 1.18 mm on 3 July 2019 (Figure 6b). The variation in shell length was best explained by model M3 (having the lowest AIC and a probability of 59% of being the best model), with substantial support for model M2 (24%) and model M4 (17%) as well (Table 4). The shell length in the raceways with a flushing rate of 600 m^3^ per tide appeared to be lower (almost 1 mm) compared to that of the other raceways (Figure 6b; Supplementary Table S3b).

On average, the shell volume increased from 717 ± 109 mm^3^ on 16 January 2019 to 4691 ± 675 mm^3^ in 3 July 2019 (Figure 6c). The variation in shell length, volume, and cockle meat content were all best explained by model M3 (54–59%), with some variation for these indices explained by M4 (15–17%) and for cockle meat content by M2 (7%) (Table 4). The shell volume in the raceways with a flushing rate of 600 m^3^ per tide appeared to be smaller (almost 800 mm^3^) compared to the raceways with different flushing rates (Figure 6c; Supplementary Table S3c).

On average, the cockle meat content increased from 0.020 ± 0.002 mg AFDW mm^−3^ on 16 January 2019 to 0.044 ± 0.003 mg AFDW mm^−3^ on 3 July 2019 (Figure 6d). The variation in condition was best explained by model M3 (having the lowest AIC and a probability of 56% of being the best model) (Table 4). The cockle meat content in the raceways with a flushing rate of 250 m^3^ and 450 m^3^ per tide were significantly smaller (more than 0.003 mg AFDW mm^−3^) than that in the raceways with flushing rates of 50 and 600 m^3^ per tide (Figure 6d; Supplementary Table S3d).

The total biomass of the cockles in the enclosures increased from 0.26 ± 0.10 mg AFDW per enclosure on 16 January 2019 to 1.21 ± 0.90 mg AFDW on 3 July 2019 (Figure 6e). The variation in condition was best explained by model M2 (having the lowest AIC and a probability of 31% of being the best model) (Table 4). The cockle total biomass in raceways 5, 7, and 13 was smaller (>0.6 mg AFDW) compared to that in raceway 1 (Figure 6e; Supplementary Table S3e).

#### 3.4.3. Experiment 2

**Figure 7:**
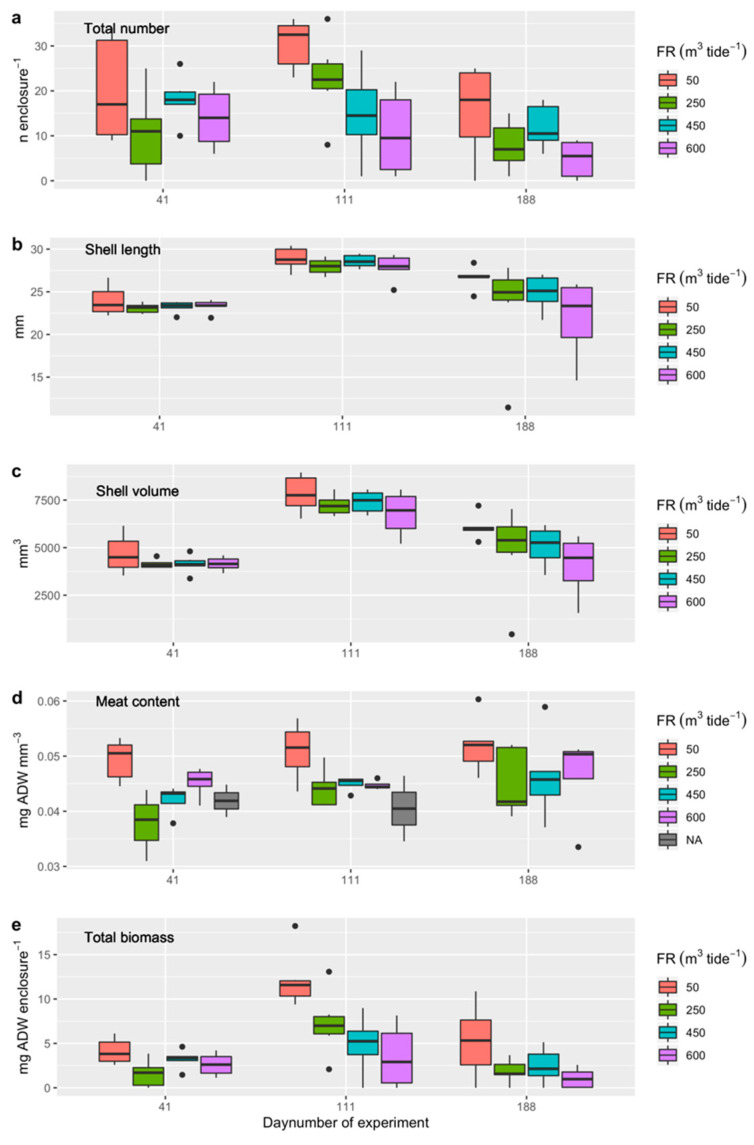
(**a**) Cockle density (numbers per enclosure), (**b**) shell length (mm), (**c**) shell volume (mm^3^), (**d**) meat content (mg AFDW mm^−3^), and (**e**) total cockle biomass (mg ADW per enclosure) for Experiment 2, where the cockles were transplanted on 6 February 2019 (day number 41), 24 April 2019 (day number 111), and 3 July 2019 (day number 188) (in 3 enclosures per raceway, with 24 enclosures per sampling period), and were all sampled on 13 August 2019 (72 enclosures in total). Day numbers on the *x*-axis are, therefore, the dates when the cockles were transplanted into the enclosures.

The number of surviving cockles sampled in August varied for the different seeding dates, with a survival of 25 ± 19% for cockles transplanted in February, 49 ± 28% for cockles transplanted in April, and 39 ± 22% for cockles transplanted in July 2019 (Figure 7a). The variation in density (number in enclosures) was best explained by model M3 (having the lowest AIC and a probability of 79% of being the best model), with substantial support for model M4 (22%) as well (Table 4). The survival in the raceways appeared to decrease with increasing flushing rates, with an average decline in the number of cockles of more than 12 individuals per enclosure for the flushing rate of 600 m^3^ per tide compared to surviving numbers in the enclosures with a flushing rate of 50 m^3^ per tide (Figure 7a; Supplementary Table S4a).

The average shell length of the cockles sampled in August was larger for the cockles seeded in April (28.33 ± 1.17 mm) than for those seeded in February (24.27 ± 4.19 mm) and July (23.41 ± 1.04 mm) (Figure 7b). The variation in shell length was best explained by model M4 (having the lowest AIC and a probability of 53% of being the best model), with substantial support for model M3 (47%) as well (Table 4). The shell length appeared to decrease with increasing flushing rates, with an average decline of almost 2 mm for the flushing rate of 600 m^3^ per tide compared to those at a flushing rate of 50 m^3^ per tide (Figure 7b; Supplementary Table S4b).

The mean shell volume of the cockles as sampled in August appeared to be higher for the cockles seeded in April (7317 ± 874 mm^3^) than for those seeded in February (5059 ± 1638 mm) and July (4285 ± 617 mm) (Figure 7c). The variation in shell volume was best explained by model H3 (having the lowest AIC and a probability of 63% of being the best model), with substantial support for model M4 (37%) as well (Table 4). The shell volume appeared to decrease with increasing flushing rates, with an average decline of more than 1300 mm^3^ for the flushing rate of 600 m^3^ per tide compared to those at a flushing rate of 50 m^3^ per tide (Figure 7c; Supplementary Table S4c).

On average, the meat content of the cockles as sampled in August appeared to be higher for the cockles seeded in February (0.048 ± 0.007 mg AFDW mm^−3^) than for those seeded in April (0.046 ± 0.005 mg AFDW mm^−3^) and July (0.044 ± 0.005 mg AFDW mm^−3^) (Figure 7d). The variation in cockle condition was best explained by model M2 (having the lowest AIC and a probability of 100% of being the best model) (Table 4). The cockle meat content was relatively high in raceway 15 (+0.011 mg AFDW mm^−3^) and relatively low in raceway 9 (−0.004 mg AFDW mm^−3^) compared to raceway 1 (Figure 7d; Supplementary Table S4d).

The total biomass of the cockles sampled in August appeared to be higher for the cockles transplanted in April (6.94 ± 4.57 mg AFDW) than for those seeded in February (3.05 ± 2.72 mg AFDW) and July (3.01 ± 1.44 mg AFDW) (Figure 7e). The variation in total biomass was best explained by model M3 (having the lowest AIC and a probability of 65% of being the best model), with substantial support for model M4 (35%) as well (Table 4). The total biomass appeared to decrease with increasing flushing rates, with an average decline of almost 5 mg AFDW for the flushing rate of 600 m^3^ per tide compared to those at flushing rate of 50 m^3^ per tide (Figure 7e; Supplementary Table S4e).

### 3.5. Correlations

The microphytobenthos concentrations (mg AFDW g^−1^) were strongly and positively correlated with the silt fraction (%) of the sediment (Table 5). With respect to the cockle performance indices, it was found that (i) the total biomass was strongly and positively correlated with the density, (ii) the shell volume (mm^3^) was strongly and positively correlated with the shell length (mm), and (iii) the shell volume (mm^3^) was positively correlated with the total biomass (mg AFDW) for both Experiment 1 and Experiment 2 (Table 5). For Experiment 2, similar positive correlations between the shell length (mm) and shell volume (mm^3^) and also between the shell length (mm) and meat content (mg AFDW mm^−3^) were found (Table 5).

For both experiments, the cockle meat content (g AFDW mm^−3^) in the raceways was positively correlated with the phytoplankton concentrations (mg CHLa m^−3^) at the entrance of the raceways (Table 5). In Experiment 1, the cockle shell length (mm) was positively correlated with the median grain size (µm) and negatively with the silt fraction (%) and microphytobenthos concentration (mg CHLa g^−1^) of the sediment (Table 5). Here, the cockle shell volume (mm^3^) and median grain size (µm) were also positively correlated (Table 5). For Experiment 2, positive correlations were found between the cockle shell length (mm) and the phytoplankton concentrations (mg CHLa m^−3^). In this experiment, negative correlations were found between the cockle volume (mm^3^) and silt fraction (%), between the cockle biomass (g AFDW) and microphytobenthos concentration (µg CHLa g^−1^), and between the cockle biomass (g AFDW) and silt fraction (%) of the sediment (Table 5).

### 3.6. Principal Component Analyses

**Figure 8:**
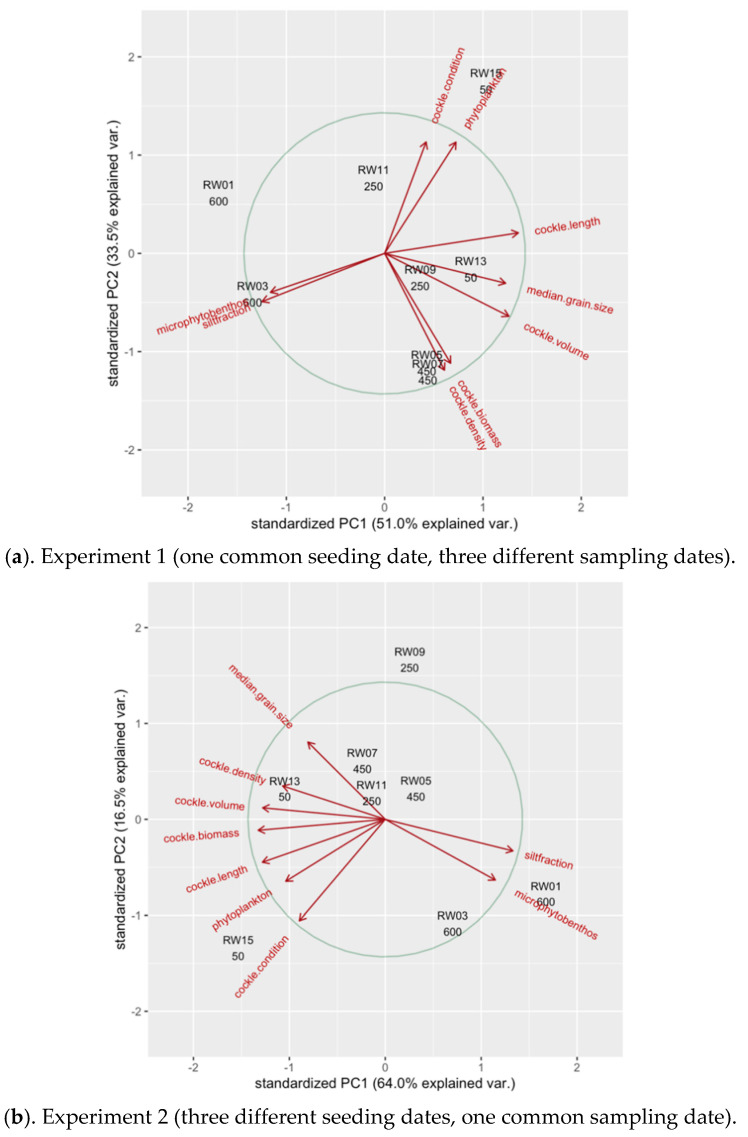
Biplots of the principal component analyses of the raceway coefficients on the environmental conditions and cockle performance indices, as derived from Models 2 (see Table 1) for (**a**) Experiment 1 and (**b**) Experiment 2.

In Experiment 1, the 1st and the 2nd axes of the PCs explained 51.0% and 33.5% of the total variance within the data set (Figure 8). The loadings of PC1 strongly suggest a gradual change in environmental conditions and cockle performance indices from RW1 to RW8 (Supplementary Figure S1). This gradient is particularly related to the variance in cockle shell length (mm), cockle shell volume (mm^3^), and median grain size (µm), being relatively large in raceways with lower flushing rates, as well as the variance in silt fraction (%) and microphytobenthos concentrations (µg AFDW g^−1^) of the sediment, which were both relatively high in raceways with higher flushing rates (Supplementary Table S5). The loadings of PC2 indicate a distinction between RW3 and RW4 (both with a flushing rate of 450 m^3^ per tide) and the other raceways (Supplementary Figure S1), in particular due to the high cockle density (number per enclosure) and total cockle biomass (g AFDW per enclosure) and the relatively low values for the cockle meat content (mg AFDW mm^−3^) and phytoplankton concentrations (mg CHLa m^−3^) in these two raceways (Supplementary Table S5).

In Experiment 2, the 1st and the 2nd axes of the PCs explained 64.0% and 16.5% of the total variance within the data set (Figure 8). As for Experiment 1, the loadings of PC1 of Experiment 2 also indicated a gradual change in the environmental conditions and cockle performance indices from RW1 to RW8 (Supplementary Figure S1). Here, this gradient is particularly related to the variance in cockle shell length (mm), cockle shell volume (mm^3^), and total cockle biomass (g AFDW per enclosure), being relatively large at raceways with lower flushing rates, as well as the variance in silt fraction (%) of the sediment, which was relatively high at raceways with higher flushing rates (Supplementary Table S5). The loadings of PC2 indicate a distinction between RW5 (with a flushing rate of 250 m^3^ per tide) and RW1 and RW8, with a flushing rate of 600 and 50 m^3^ per tide, respectively (Supplementary Figure S1). This difference was particularly due to a relatively large median grain size (µm) and a relatively low cockle meat content (mg AFDW mm^−3^) in this raceway compared to the two others (Supplementary Table S5).

## 4. Discussion

### 4.1. Ecological Engineering of Environmental Conditions

The results of the environmental conditions strongly suggest that increasing the residence time of seawater before supplying it to the cockles resulted in the sedimentation of the silt content of the incoming seawater and in strengthening and enhancing the spring bloom of the phytoplankton from mid-April (>10 mg CHLa m^−3^ in RW5, 9, 10, 11, and 13) to as early as the end of January (>10 mg CHLa m^−3^ in RW11 and 13). Because the growth of pelagic microalgae is light-limited in early spring [32], the enhanced timing of the spring bloom was probably the result of a decrease in the turbidity. Such an advanced bloom could then also take advantage of the high concentrations of nutrients after the winter [33,34], subsequently resulting in a high productivity and high biomass of phytoplankton. We cannot exclude, however, that the increased intensity of the blooms during the rest of the growing season was at least partly due to the additional nutrient supply from the older sediments.

If the inflowing silt concentrations in the water were similar for all raceways, the raceways with high flushing rates (600 m^3^ per tide) would have experienced 12 times higher silt import rates than the ones with the low flushing rates (50 m^3^ per tide). However, based on the observations of the phytoplankton concentrations as well as the contribution of the distance to inlet to the silt fraction in the raceways, the silt concentrations in the water entering the raceways were lower as the raceways were located further downstream. This implies that the differences in net import (silt volume per tide) were even larger between raceways with high (600 m^3^ per tide) compared to low (50 m^3^ per tide) flushing rates.

The net sedimentation of the silt content of the incoming seawater is the difference between the import and export of this material [35]. If there were only import and no export of silt, the silt fraction would have been accumulating during the full experimental period. However, after an initial period of silt accumulation from December 2018 to April 2019, the silt fraction in the raceways remained more or less constant until July 2019 and subsequently decreased. This implies that silt export also must have occurred. This seasonal pattern of sediment composition resembles the natural seasonal dynamics on tidal flats where silt accumulates during the spring and summer and then washes away due to wave-generated resuspension [36].

The silt content of the sediment was strongly and positively correlated with the concentrations of microphytobenthos. On tidal flats, the sediment type and bathymetry are considered as the main factors determining the spatial variation in the biomass of microphytobenthos [37,38]. The observed correlation with sediment type is generally explained by the microphytobenthos growth, promoted by low dynamic energy, as reflected by the high silt contents of the sediment [39,40]. However, the highest silt and microphytobenthos concentrations in the aquaculture system were observed in the raceways which experienced the highest (but still low) flushing rates. Most likely, a positive feedback occurred here between the net silt accumulation and diatom growth due to the relatively high concentrations of nutrients in silt-rich sediment compared to more sandy sediments, as was also observed for tidal flats [40].

In summary, the combination of the increased distance to tidal inlet (+75 m) and reduced flushing rates (from 600 to 50 m^3^ per tide) resulted in higher spring blooms of phytoplankton (+3 mg CHLa m^−3^) occurring much earlier in the season (weeks to months) in the reduced silt fraction of the sediment (−13%) and in the lower standing stocks of microphytobenthos (−5.8 µg CHLa g^−1^).

### 4.2. Impacts on Cockle Performance Indices

The maximum number of cockles in the experiments was 9 × 40 = 360 individuals per raceway, implying a maximum density of 2.4 individuals per m^2^. With a minimum flushing rate of 50 m^3^ per tide and an average depth of 0.3 m, the water renewal rate was 1.1 m^3^ per m^2^ per tide. Assuming a pumping rate of an adult cockle of 1.3 dm^3^ h^−1^ [41], 2.4 cockles m^−2^ would pump 0.02 m^3^ per tide, which is less than 2% of the water renewal rate. This means that food supply was not likely to be limiting during the experiments, and that the observed differences in cockle performance were more likely to be due to the quality of the food and/or growth-restricting environmental conditions.

The survival was relatively high in the raceways with intermediate flushing rates (250 m^3^ per tide in Experiment 1, 450 m^3^ per tide in Experiment 2), and unrelated to the gradients in distance to tidal inlet (raceways 1 to 8). Apparently, mortality was determined by a local factor (e.g., predation), which was not included in the sampling scheme of the environmental conditions. On average, the survival was highest (68%) for the cockles that were seeded at the end of April and sampled in mid-August 2019, and lowest (12%) for cockles transplanted mid-December 2018 and sampled in July 2019. These survival rates are within the range of the (strongly variable) annual survival rates of cockles on nearby tidal flats [42]. For Experiment 2, cockle survival was apparently related to the quality of the stock used for these transplants. This implies that a good stocking material is crucial for acquiring high survival rates during inshore culturing.

For both experiments, the shell length (mm), shell volume (mm^3^), and meat content (µg AFDW mm^3^) appeared to be promoted by high phytoplankton concentrations and hampered by high microphytobenthos concentrations. The positive correlation between cockle growth and phytoplankton concentrations is in line with previous findings from field observations and (transplant) experiments [43,44]. The growth reduction in the cockles in raceways which were relatively rich in microphytobenthos is, however, in contrast with other findings [45].

Most probably, the surplus of food sources is overruled by the restricted grazing due to the high silt contents of the sediment, as was also observed for cockles in waters with high (>40 g DW m^−3^) silt concentrations [46] and sediments of tidal flats with high silt fractions [47]. This suggests that the supply of seawater with a high phytoplankton concentration and a low silt content favors cockle growth, and that cockles cannot profit from a high concentration of benthic microalgae when these grow in silty sediments.

### 4.3. Future Requirements and Perspectives

The results of the experiments showed that the growth conditions (with respect to water and sediment quality) for the inshore cultivation of cockles can be improved by means of ecological engineering. The design and maintenance of an inshore culturing facility to optimize growth conditions will depend on the temperature and composition of the seawater (with respect to the nutrients, phytoplankton, and silt content), the size and meat content of the seeding material, the intended yield (number of cockles per year of commercial size), and the surface area of the cockle fields. Assuming a commercially interesting yield of 500 marketable cockles per m^2^ (as estimated by members of the shellfish industry) and an individual cockle pumping rate of 1.3 dm^3^ h^−1^ [41] for 6 h per tide, then the renewal rate should be around 4 m^3^ per m^2^ per tide (being 40.000 m^3^ per hectare).

Furthermore, controlling the inflow and outflow of the seawater can aid in protecting cockles against unfavorable conditions. Keeping the cockles submerged during extreme low and high temperatures will prevent mass mortality, as has been observed in the field during severe winters and the recent heat waves [12]. Restricting the inflow when the seawater is brackish (after downpours and subsequent strong river run-off; [48]) or very turbid (following severe storms in shallow coastal waters [49]) will limit the energy spent by the cockles to withstand such conditions [46] and subsequently result in better growth and survival. When bordering marine waters with variable water quality during the tidal cycle (e.g., the Wadden Sea water during outgoing tide is much more turbid than during the incoming tide), managing when water enters the facility may further improve the yield of the inshore cockle farm.

The growth and survival of the cockles within the aquaculture system appeared to be strongly related to the origin of the juvenile cockles that were seeded, including the timing when they were put into the raceways. This implies that the final yield of a cockle farm will not only depend on the conditions created within the raceways but also on the quality of the starting material. Assuming a mortality of 50% during farming, the starting density should be 1000 juveniles m^−2^ (being 10,000,000 juveniles per hectare). Juvenile cockles can be derived from fisheries or from hatcheries. While sourcing juvenile cockles from hatcheries is possible [50], a grow-out production system that would reduce larval mortality is very costly, rendering such a practice not economically sustainable. Because the recruitment success of natural stocks varies strongly from year to year [51,52], the supply from fisheries might be too unpredictable for the required annual yield.

Recruitment failure is considered to be predominantly due to temperature-related high predation rates on post-larvae [53,54] and/or high adult densities inhibiting post-larval settlement [55,56]. If so, nurseries could be set up with bare sediments and devoid of predatory shrimps and crabs, allowing successful settlement of larvae released from well-conditioned adult cockle stocks. Because a female cockle may produce up to 700,000 eggs [57], it should be possible in theory to make cockle farms self-sufficient by rotating spawning stock areas, nurseries and harvesting grounds. Further increases in the yield could then be accomplished by selecting cockles for the parent stock which show a fast growth and a high survival rate within the environmental conditions of the aquaculture system [58]. To become independent from outside supply, and also do not affect the natural population, further research should be towards obtaining the settlement by self-reproduction of an inshore stock.

In addition to the increasing human demands for seafood, the recent observations on declining aquaculture yields of mussels at a European scale [59] and the growing shortage in juvenile oyster supply for aquaculture at a global scale [60] further stresses the need for the exploration of the potential of the self-sustaining and sustainable cultivation of coastal shellfish. Maximizing yields and minimizing (environmental) costs have to be targeted when selecting the best locations, developing the best design, and optimizing the operational procedures of inshore marine shellfish farms.

With respect to costs, we foresee that energy use is relatively low, as water renewal is driven by the tidal amplitude. Annual costs will include the maintenance of the system (e.g., operation and maintenance of the waterflows, removing the surplus of sediment, keeping the cockles free from macroalgae and predators), the sampling and seeding of the juvenile cockles, harvesting. and depuration (which is presently done for wild cockles by flushing them for 2–3 days in the processing factory with saline groundwater). These costs (in Euro per hectare per year) depend on the design of the farm, local environmental conditions, the size of the farm, and local labor costs. Because the inshore bivalves graze upon the natural supply of marine microalgae, no additional costs have to be made for food.

With respect to returns, these very much rely on the shell length and density at harvesting. Cockles with a minimum shell length of 27 mm can be sold as a fresh product at a price of approximately 2 Euro per kg (including shells). Based upon a conversion of 100 cockles of that size per kg and assuming a density of 500 harvestable cockles per m^2^, the returns would be 100,000 Euro per hectare. Smaller cockles with a shell length between 24 and 27 mm can be cooked and canned, and subsequently sold at a price of 6 Euro per kg (meat only). Based upon a conversion of 250 cockles of that size per kg, a meat content of 17% and assuming a density of 500 harvestable cockles per m^2^, the returns would be just over 20,000 Euro per hectare. Stimulating the fast growth of the cockles to a shell length of 27 mm is, therefore, crucial to make inshore cockle aquaculture commercially attractive.

With respect to feasibility, this relies, amongst others, on the permission to build a cockle farm. Within the Netherlands, cockle farms are presently considered as a possible solution to combat sea level rise in low-lying areas. By building a second dike inland (instead of re-enforcing the seaward dike), the hinterland is protected against storm surges whilst the land between the two dikes can be used for inshore marine aquaculture of shellfish. This innovative and cost-effective approach combines water safety with sustainable economic opportunities.

As the cockles should also be harvested and marketed, which requires knowledge on and experience of best practices, the success of such a system strongly relies on further collaboration between researchers and stakeholders from the shellfish fishery industry to get a full grip on the costs and returns of inshore estuarine shellfish farms.

## 5. Conclusions

Declining marine fishing yields, due to overfishing and the impacts of climate change, hamper the increasing demand for healthy and nutritious seafood for a growing human population. Inshore shellfish farming may be an innovative and sustainable means of seafood production, securing a stable long-term food supply with minimum ecological impacts. Our experiments in an inshore testing site revealed that the growth conditions for the inshore cultivation of suspension-feeding shellfish can indeed be improved by means of nature-based ecological engineering, where proper design and flushing rates enhance the water quality (more phytoplankton growth, lower silt concentrations). Cockle growth and survival can be further strengthened by keeping the shellfish submerged during extreme weather conditions (ice winters, heat waves), and by creating a continuous natural supply of high-quality recruits within the cockle farm. These results underline the potential of a global transition towards innovative land-based mariculture, in particular for salinized coastal lowlands where the yield by common agriculture practices is reduced. The next steps include the identification of optimal locations for such practices, testing the consequences of design and operation at commercially interesting scales, and ensuring a continuous supply of high-quality recruits to stock mariculture systems.

## Figures and Tables

**Table 1:** Hypotheses (H) and statistical models (M) for the variation in environmental conditions and cockle performance indices during the experiment from 12 December 2018 to 1 August 2019. Seasonality (*i*) is based upon 15 (for phytoplankton) and 3 (for all other variables) sampling dates. The raceways (*j*) were numbered from 1 to 8, the flushing rates (*k*) were 50, 250, 450, and 600 m^3^ per tide. Models with smoothers could be applied for the data on phytoplankton concentrations only.

#	Hypothesis	Statistical Model			
		Factors		Smoothers	
H1	There is one similar seasonal pattern for all raceways	M1f	y_i_ = ß_i_ + SamplingDate_i_ + E_i_	M1s	y_i_ = ß_i_ + s_i_(SamplingDate) + E_i_
H2	There is one seasonal pattern, with an additional effect of raceway	M2f	y_ij_ = ß_i_ + SamplingDate_i_ + RaceWay_j_ + E_ij_	M2s	y_i_ = ß_i_ + s_i_(SamplingDate) + RaceWay_j_ + E_ij_
H3	There is one seasonal pattern, with an additional effect of flushing rate	M3f	y_ij_ = ß_i_ + SamplingDate_i_ + FlushRate_k_ + E_ik_	M3s	y_i_ = ß_i_ + s_i_(SamplingDate) + Flushrate_k_ + E_ik_
H4	There is one seasonal pattern, with the additional effects of flushing rate and relative distance to inlet	M4f	y_i_ = ß_i_ + SamplingDate_i_ + FlushRate_k_ + RelDist_l_ + E_ikl_	M4s	y_i_ = ß_i_ + s_i_(SamplingDate) + FlushRate_k_ + RelDist_l_ + E_ikl_
H5	There are different seasonal patterns per raceway			M5s	y_i_ = ß_i_ + s_ij_(SamplingDate, RaceWay) + E_ij_
H6	There are different seasonal patterns per flushing rate			M6s	y_i_ = ß_i_ + s_ik_(SamplingDate, FlushRate) + E_ik_
H7	There are different seasonal patterns per flushing rate, with an additional effect of relative distance to inlet			M7s	y_i_ = ß_i_ + s_ik_(SamplingDate, FlushRate) + RelDist_l_ + E_ikl_

**Table 2:** Values and differences in the Akaike Information Criterion (AIC) and the Akaike weight (*w_i_*) for statistical models (M1–M7 in Table 1) for the phytoplankton biomass. The model with the best fit (for which AIC has the lowest value) is printed in bold.

Model	Type	Microalgal Biomass		
		Phytoplankton		
		AIC	Δ*i*	*w_i_*
M1	Factors	643.7	62.3	0.00
	Smoothers	649.1	67.7	0.00
M2	Factors	623.4	42.0	0.00
	Smoothers	633.7	52.3	0.00
M3	Factors	628.9	47.5	0.00
	Smoothers	637.1	55.7	0.00
M4	Factors	627.6	46.2	0.00
	Smoothers	636.3	54.9	0.00
M5	Smoothers	**581.4**	0.0	**1.00**
M6	Smoothers	658.9	77.4	0.00
M7	Smoothers	658.109	76.7	0.00

**Table 3:** Values and differences of the Akaike Information Criterion (AIC), and the Akaike weight (*w_i_*) for statistical models (M1–M4 in Table 1) for the sediment characteristics and microphytobenthic biomass. Models for which the level of empirical support for model *i* is substantial (i.e., the value of Δ*i* is between 0 and 2) are underlined, and the best fit (for which AIC has the lowest value) is printed in bold.

Model	Sediment Characteristics						Microalgal Biomass		
	Median Grain Size			Silt Fraction			Microphytobenthos		
	Factors			Factors			Factors		
	AIC	Δ*i*	*w_i_*	AIC	Δ*i*	*w_i_*	AIC	Δ*i*	*w_i_*
M1	1962.8	35.3	0.00	1520.6	101.2	0.00	1220.5	89.8	0.00
M2	1931.8	4.3	0.05	1424.2	4.8	0.04	1135.1	4.5	0.04
M3	**1927.5**	**0.0**	**0.71**	1421.6	2.2	0.26	**1130.7**	**0.0**	**0.65**
M4	1928.5	1.0	0.24	**1419.4**	**0.0**	**0.70**	1132.0	1.3	0.31

**Table 4:** Values and differences for the Akaike Information Criterion (AIC), and the Akaike weight (*w_i_*) for statistical models (M1–M4 in Table 1) for the cockle performance indices. Models for which the level of empirical support for model *i* is substantial (i.e., the value of Δ*i* is between 0 and 2) are underlined, and the best fit (for which AIC has the lowest value) is printed in bold.

Index	Model	Experiment 1			Experiment 2		
		AIC	Δ*i*	*w_i_*	AIC	Δ*i*	*w_i_*
Density	M1	449.4	15.3	0.00	530.0	54.9	0.00
	M2	**434.1**	**0.0**	**0.39**	503.7	28.6	0.00
	M3	436.4	2.3	0.47	**475.1**	**0.0**	**0.79**
	M4	438.4	4.2	0.14	477.1	2.0	0.22
Shell length	M1	177.7	3.1	0.24	322.5	60.8	0.00
	M2	182.2	7.5	0.00	325.7	64.0	0.00
	M3	**174.7**	**0.0**	**0.59**	262.5	0.9	0.47
	M4	176.6	2.0	0.17	**261.7**	**0.0**	**0.53**
Shell volume	M1	1228.6	2.5	0.28	1266.5	108.6	0.00
	M2	1231.8	5.7	0.01	1267.6	109.7	0.00
	M3	**1226.1**	**0.0**	**0.54**	**1157.9**	**0.0**	**0.63**
	M4	1227.9	1.8	0.17	1158.3	0.5	0.37
Meat content	M1	−199.6	3.5	0.22	−174.1	43.9	0.00
	M2	−202.8	0.3	0.07	**−218.0**	**0.0**	**1.00**
	M3	**−203.1**	**0.0**	**0.56**	−179.9	38.1	0.00
	M4	−201.2	2.0	0.15	−193.1	24.9	0.00
Total biomass	M1	467.0	9.3	0.02	708.4	78.0	0.00
	M2	**457.7**	**0.0**	**0.31**	687.7	57.3	0.00
	M3	460.2	2.5	0.34	**630.4**	**0.0**	**0.65**
	M4	459.8	2.1	0.32	631.0	0.7	0.35

**Table 5:** Correlations and significance of correlations between the environmental condition and cockle performance indices. Significant correlations (*p* < 0.05) are printed in bold, correlations with *p*-values < 0.1 are underlined. To aid comparison between experiments, cells with positive correlations with *p*-values smaller than 0.1 are colored blue and cells with negative correlations with *p*-values smaller than 0.1 are colored orange. Significance values were not corrected for the multiple comparisons.

Group	Variable 1	Variable 2	Experiment 1		Experiment 2	
			R	*p*	R	*p*
Environmental conditions	Phytoplankton	Microphytobenthos	−0.479	0.229	Similar to Experiment 1	
		Median grain size	0.239	0.569		
		Silt fraction	−0.643	0.085		
	Microphyto-benthos	Median grain size	−0.604	0.113		
		Silt fraction	**0.960**	**0.000**		
	Median grain size	Silt fraction	−0.600	0.116		
Cockle performance indices	Density	Shell length	0.312	0.452	0.544	0.164
		Shell volume	**0.714**	**0.047**	0.675	0.066
		Condition	−0.397	0.330	0.268	0.520
		Total biomass	**0.945**	**0.000**	**0.878**	**0.004**
	Shell length	Shell volume	**0.743**	**0.035**	**0.877**	**0.004**
		Meat content	0.481	0.228	**0.813**	**0.014**
		Total biomass	0.382	0.350	**0.791**	**0.019**
	Shell volume	Condition	−0.132	0.755	0.521	0.186
		Total biomass	**0.747**	**0.033**	**0.760**	**0.029**
	Meat content	Total biomass	−0.278	0.505	0.596	0.119
Cross correlations	Cockle density	Phytoplankton	−0.327	0.429	0.233	0.579
		Microphytobenthos	0.014	0.973	−0.641	0.087
		Median grain size	0.474	0.236	0.288	0.489
		Silt fraction	−0.046	0.913	−0.691	0.058
	Cockle length	Phytoplankton	0.633	0.092	**0.730**	**0.040**
		Microphytobenthos	**−0.714**	**0.047**	−0.493	0.215
		Median grain size	**0.810**	**0.015**	0.457	0.255
		Silt fraction	**−0.804**	**0.016**	−0.696	0.055
	Cockle volume	Phytoplankton	0.062	0.883	0.495	0.212
		Microphytobenthos	−0.646	0.084	−0.620	0.101
		Median grain size	**0.843**	**0.009**	0.658	0.076
		Silt fraction	−0.648	0.082	**−0.760**	**0.029**
	Cockle meat content	Phytoplankton	**0.931**	**0.001**	**0.719**	**0.045**
		Microphytobenthos	−0.207	0.622	−0.172	0.685
		Median grain size	0.039	0.928	−0.023	0.957
		Silt fraction	−0.392	0.337	−0.418	0.303
	Cockle biomass	Phytoplankton	−0.277	0.506	0.663	0.073
		Microphytobenthos	−0.016	0.969	**−0.710**	**0.049**
		Median grain size	0.471	0.238	0.293	0.481
		Silt fraction	−0.071	0.868	**−0.834**	**0.010**

## Supplementary Materials

The following are available online at https://www.mdpi.com/1660-4601/17/19/7224/s1, Table S1. Number of samples taken during the experiments. Table S2: The estimates, estimated degrees of freedom and approximate significance for intercepts and smoothers of the best statistical model for phytoplankton, microphytobenthos, median grain size and silt fraction. Table S3: The estimates, their standard error and the significance for intercepts and factors of the best statistical models for cockle density, shell length, shell volume, meat content and total cockle biomass of Experiment 1. Table S4: The estimates, their standard error and the significance for intercepts and factors of the best statistical models for cockle density, shell length, shell volume, meat content and total cockle biomass of Experiment 2. Table S5: Scores of the PCA of Experiment 1 and Experiment 2. Figure S1. Loadings of PCA.
