# Supplementary material for: Ecological Engineering for the Optimisation of the Land-Based Marine Aquaculture of Coastal Shellfish
Source: Int J Environ Res Public Health. 2020 Oct 2;17(19):7224. doi: 10.3390/ijerph17197224 (PMC7579225; doi:10.3390/ijerph17197224)
Supplement: Supplementary file 1 [file ijerph-17-07224-s001.pdf]

SUPPLEMENTARY MATERIALS

A. Number of samples taken during the experiments (Table S1)

Table S1. Number of samples taken during the experiments. Phytoplankton (PHYT): one sample per raceway was taken. Microphytobenthos (MPB) and sediment characteristics (SED): three enclosures per raceway. At the start of the experiment (t_0), samples for microphytobenthos and sediment characteristics were taken from all enclosures (9 per raceway). For Experiment 1, in total 772 living cockles were sampled from the enclosures, which was almost 27% of the maximum number if all cockles in this experiment had survived during the experiment (being 72x40 = 2880 individuals). For Experiment 2, 1091 living cockles were sampled from the enclosures, which is almost 38% of the maximum number if all cockles in this experiment had survived during the experiment.

**Date**	**Day nr**	**Environmental conditions**			**Cockle performance indices**			
		**PHYT**	**MPB**	**SED**	**Experiment 1**		**Experiment 2**	
		sampled	sampled	sampled	seeded	sampled	*seeded*	sampled
**2018-12-12**	0		72	72	72			
2019-01-16	35		24	24		24		
2019-01-22	41	8						
2019-02-05	55	8						
2019-02-06	56						*24*	
2019-02-19	69	8						
2019-03-05	86	8						
2019-03-19	100	8						
2019-04-02	113	8						
2019-04-16	127	8						
2019-04-24	133		24	24		24	*24*	
2019-04-30	139	8						
2019-05-14	153	8						
2019-05-28	167	8						
2019-06-11	181	8						
2019-06-25	195	8						
2019-07-03	203		24	24		24	*24*	
2019-07-09	209	8						
2019-07-23	223	8						
2019-08-06	238	8						
2019-08-13	245		72	72				72
**SUM**		**120**	**216**	**216**	**72**	**72**	**72**	**72**

B. RESULTS STATISTICAL ANALYSES

Table S2. The estimates, estimated degrees of freedom and approximate significance for intercepts and smoothers of the best statistical model for **(a)** phytoplankton (mg CHLa m^{-3}), **(b)** microphytobenthos (µg CHLa g^{-1}), **(c)** median grain size (µm) and **(d)** silt fraction (%) (see Table 1 for description of models). Significance codes: '***' < 0.001,'**' < 0.01, '*'< 0.05 and '.' < 0.1.

a. Phytoplankton

```
Formula:
CHLa ~ s(Tid, RW)

Parametric coefficients:
            Estimate Std. Error t value Pr(>|t|)
(Intercept)    6.729      0.224   30.04   <2e-16 ***

Approximate significance of smooth terms:
            edf Ref.df     F p-value
s(Tid,RW) 26.04  28.42 8.841  <2e-16 ***

R-sq.(adj) =  0.668   Deviance explained =   74%
GCV = 7.7724  Scale est. = 6.021     n = 120
```

b. Microphytobenthos

```
Formula:
CHLa ~ factor(SamplingDate) + factor(RefVol)

Parametric coefficients:
                              Estimate Std. Error t value Pr(>|t|)
(Intercept)                    -0.4789     0.5400  -0.887   0.3761
factor(SamplingDate)2019-01-16  1.4155     0.7637   1.854   0.0652 .
factor(SamplingDate)2019-04-24  7.8177     0.7637  10.237  < 2e-16 ***
factor(SamplingDate)2019-07-03  7.5824     0.7637   9.929  < 2e-16 ***
factor(SamplingDate)2019-08-13  4.7042     0.5400   8.712 9.41e-16 ***
factor(RefVol)250               0.1069     0.6235   0.171   0.8640
factor(RefVol)450               3.0515     0.6235   4.894 1.98e-06 ***
factor(RefVol)600               5.7532     0.6235   9.227  < 2e-16 ***

R-sq.(adj) =  0.577   Deviance explained = 59.1%
GCV = 10.901  Scale est. = 10.497    n = 216
```

c. Median grain size

```
Formula:
MedGS ~ factor(SamplingDate) + factor(RefVol)

Parametric coefficients:
                              Estimate Std. Error t value Pr(>|t|)
(Intercept)                    225.288      3.415  65.961  < 2e-16 ***
factor(SamplingDate)2019-01-16  -9.571      4.830  -1.981   0.0489 *
factor(SamplingDate)2019-04-24 -31.859      4.830  -6.596 3.44e-10 ***
factor(SamplingDate)2019-07-03 -30.717      4.830  -6.359 1.26e-09 ***
factor(SamplingDate)2019-08-13 -19.176      3.415  -5.615 6.26e-08 ***
factor(RefVol)250                1.665      3.944   0.422   0.6734
factor(RefVol)450                7.159      3.944   1.815   0.0709 .
factor(RefVol)600              -17.476      3.944  -4.431 1.51e-05 ***

R-sq.(adj) =  0.339   Deviance explained =   36%
GCV = 436.11  Scale est. = 419.96    n = 216
```

d. Silt fraction (%)

```
Formula:
Vol63 ~ factor(SamplingDate) + factor(RefVol) + factor(RWrank)

Parametric coefficients:
                               Estimate Std. Error t value Pr(>|t|)    
(Intercept)                     -0.2402     1.1354  -0.212  0.83268    
factor(SamplingDate)2019-01-16   6.7972     1.4866   4.572 8.30e-06 ***
factor(SamplingDate)2019-04-24  14.6397     1.4866   9.848  < 2e-16 ***
factor(SamplingDate)2019-07-03  13.4206     1.4866   9.028  < 2e-16 ***
factor(SamplingDate)2019-08-13   9.2647     1.0512   8.813 4.98e-16 ***
factor(RefVol)250                3.5013     1.2138   2.885  0.00433 ** 
factor(RefVol)450                7.5726     1.2138   6.239 2.45e-09 ***
factor(RefVol)600               13.2517     1.2138  10.917  < 2e-16 ***
factor(RWrank)Upstream           1.7348     0.8583   2.021  0.04454 *  

R-sq.(adj) =  0.571   Deviance explained = 58.7%
GCV =  41.51  Scale est. = 39.781    n = 216
```

Table S3. The estimates, their standard error and the significance for intercepts and factors of the best statistical models for **(a)** cockle density (number per enclosure), **(b)** shell length (mm), **(c)** shell volume (mm^3), **(d)** meat content (mg ADW mm^{-3}) and **(e)** total cockle biomass (mg ADW per enclosure) of Experiment 1 (see Table 1 for descriptions of the models). Significance codes: '***' < 0.001,'**' < 0.01, '*'< 0.05 and '.' < 0.1.

a. Density

Formula:
Tn ~ factor(SamplingDate) + factor(RW)

Parametric coefficients:

	Estimate	Std. Error	t value	Pr(>|t|)	
(Intercept)	14.5972	1.7002	8.586	3.84e-12	***
factor(SamplingDate)2019-04-24	-10.1250	1.3170	-7.688	1.38e-10	***
factor(SamplingDate)2019-07-03	-13.6667	1.3170	-10.377	3.50e-15	***
factor(RW)3	4.5556	2.1506	2.118	0.038169	*
factor(RW)5	7.6667	2.1506	3.565	0.000708	***
factor(RW)7	8.7778	2.1506	4.082	0.000130	***
factor(RW)9	3.3333	2.1506	1.550	0.126245	
factor(RW)11	0.5556	2.1506	0.258	0.797013	
factor(RW)13	5.8889	2.1506	2.738	0.008052	**
factor(RW)15	2.1111	2.1506	0.982	0.330097	

R-sq.(adj) = 0.66 Deviance explained = 70.4%
GCV = 24.17 Scale est. = 20.813 n = 72

b. Shell length

Formula:
Tl ~ factor(SamplingDate) + factor(RW)

Parametric coefficients:

	Estimate	Std. Error	t value	Pr(>|t|)	
(Intercept)	12.3886	0.3524	35.156	<2e-16	***
factor(SamplingDate)2019-04-24	4.9406	0.2503	19.741	<2e-16	***
factor(SamplingDate)2019-07-03	11.5448	0.2682	43.046	<2e-16	***
factor(RW)3	-0.1766	0.4494	-0.393	0.6959	
factor(RW)5	0.6013	0.4392	1.369	0.1764	
factor(RW)7	0.4968	0.4392	1.131	0.2627	
factor(RW)9	0.3874	0.4392	0.882	0.3815	
factor(RW)11	0.3257	0.4494	0.725	0.4716	
factor(RW)13	0.6745	0.4392	1.536	0.1302	
factor(RW)15	0.8549	0.4494	1.902	0.0622	.

R-sq.(adj) = 0.966 Deviance explained = 97.1%
GCV = 0.88348 Scale est. = 0.75162 n = 67

c. Shell volume

Formula:
Tv ~ factor(SamplingDate) + factor(Vol)

Parametric coefficients:

	Estimate	Std. Error	t value	Pr(>|t|)	
(Intercept)	935.3	330.2	2.833	0.006111	**
factor(SamplingDate)2019-04-24	1180.7	330.2	3.576	0.000658	***
factor(SamplingDate)2019-07-03	2996.0	330.2	9.075	3.21e-13	***
factor(Vol)250	-146.9	381.2	-0.385	0.701172	
factor(Vol)450	139.7	381.2	0.366	0.715292	
factor(Vol)600	-864.6	381.2	-2.268	0.026613	*

R-sq.(adj) = 0.55 Deviance explained = 58.2%
GCV = 1.4269e+06 Scale est. = 1.308e+06 n = 72

d. Meat content

```
Formula:
Tb ~ factor(SamplingDate) + factor(Vol)

Parametric coefficients:
                              Estimate Std. Error t value Pr(>|t|)    
(Intercept)                   0.022354   0.001087  20.574  < 2e-16 ***
factor(SamplingDate)2019-04-24  0.016728   0.001106  15.119  < 2e-16 ***
factor(SamplingDate)2019-07-03  0.023909   0.001182  20.227  < 2e-16 ***
factor(Vol)250               -0.003328   0.001451  -2.294  0.02621 *  
factor(Vol)450               -0.003589   0.001258  -2.853  0.00637 ** 
factor(Vol)600               -0.002693   0.001362  -1.977  0.05382 .  
---
Signif. codes:  0 '***' 0.001 '**' 0.01 '*' 0.05 '.' 0.1 ' ' 1


R-sq.(adj) =    0.9   Deviance explained =   91%
GCV = 1.3294e-05  Scale est. = 1.1817e-05  n = 54
```

e. Total biomass

```
Formula:
Tw ~ factor(SamplingDate) + factor(RW)

Parametric coefficients:
                              Estimate Std. Error t value Pr(>|t|)    
(Intercept)                   -0.07951    0.20029  -0.397  0.69276    
factor(SamplingDate)2019-04-24  0.26705    0.15515   1.721  0.09019 .  
factor(SamplingDate)2019-07-03  0.69844    0.15515   4.502 3.03e-05 ***
factor(RW)3                    0.29886    0.25335   1.180  0.24266    
factor(RW)5                    0.72743    0.25335   2.871  0.00559 ** 
factor(RW)7                    0.67567    0.25335   2.667  0.00975 ** 
factor(RW)9                    0.38385    0.25335   1.515  0.13483    
factor(RW)11                  -0.16786    0.25335  -0.663  0.51007    
factor(RW)13                   0.63163    0.25335   2.493  0.01535 *  
factor(RW)15                   0.15551    0.25335   0.614  0.54158    


R-sq.(adj) =  0.332   Deviance explained = 41.7%
GCV = 0.33544  Scale est. = 0.28885   n = 72
```

Table S4. The estimates, their standard error and the significance for intercepts and factors of the best statistical models for **(a)** cockle density (number per enclosure), **(b)** shell length (mm), **(c)** shell volume (mm^3), **(d)** meat content (mg ADW mm^{-3}) and **(e)** total cockle biomass (mg ADW per enclosure) of Experiment 2 (see Table 1 for descriptions of the models). Significance codes: '***' < 0.001,'**' < 0.01, '*'< 0.05 and '.' < 0.1.

a. Density

Formula:
Tn ~ factor(SeedingDate) + factor(RW)

Parametric coefficients:

	Estimate	Std. Error	t value	Pr(>|t|)	
(Intercept)	1.264	2.757	0.458	0.648265	
factor(SeedingDate)2019-04-24	9.583	2.136	4.487	3.19e-05	***
factor(SeedingDate)2019-07-03	5.625	2.136	2.634	0.010647	*
factor(RW)3	6.889	3.488	1.975	0.052692	.
factor(RW)5	4.889	3.488	1.402	0.165958	
factor(RW)7	12.556	3.488	3.600	0.000633	***
factor(RW)9	8.111	3.488	2.326	0.023319	*
factor(RW)11	6.556	3.488	1.880	0.064849	.
factor(RW)13	21.667	3.488	6.213	4.85e-08	***
factor(RW)15	9.889	3.488	2.835	0.006171	**

R-sq.(adj) = 0.449 Deviance explained = 51.9%
GCV = 63.561 Scale est. = 54.733 n = 72

b. Shell length

Formula:
Tl ~ factor(SeedingDate) + factor(RW)

Parametric coefficients:

	Estimate	Std. Error	t value	Pr(>|t|)	
(Intercept)	22.6518	1.0775	21.022	< 2e-16	***
factor(SeedingDate)2019-04-24	4.1252	0.7350	5.612	5.88e-07	***
factor(SeedingDate)2019-07-03	-0.7950	0.7441	-1.068	0.28974	
factor(RW)3	1.3271	1.2375	1.072	0.28799	
factor(RW)5	1.4879	1.2375	1.202	0.23410	
factor(RW)7	2.1539	1.2375	1.741	0.08707	.
factor(RW)9	0.5183	1.2375	0.419	0.67687	
factor(RW)11	1.5489	1.2743	1.215	0.22912	
factor(RW)13	2.0021	1.2375	1.618	0.11112	
factor(RW)15	3.4139	1.2664	2.696	0.00918	**

R-sq.(adj) = 0.452 Deviance explained = 52.6%
GCV = 6.9992 Scale est. = 5.9699 n = 68

c. Shell volume

```
Formula:
Tv ~ factor(SeedingDate) + factor(RW)

Parametric coefficients:
                              Estimate Std. Error t value Pr(>|t|)
(Intercept)                     3025.6      554.9   5.453 9.15e-07 ***
factor(SeedingDate)2019-04-24   2890.6      429.8   6.725 6.41e-09 ***
factor(SeedingDate)2019-07-03   -320.5      429.8  -0.746  0.45868
factor(RW)3                     1321.7      701.9   1.883  0.06438 .
factor(RW)5                     1469.7      701.9   2.094  0.04036 *
factor(RW)7                     1855.0      701.9   2.643  0.01039 *
factor(RW)9                     1363.3      701.9   1.942  0.05664 .
factor(RW)11                    1237.5      701.9   1.763  0.08280 .
factor(RW)13                    1746.0      701.9   2.488  0.01556 *
factor(RW)15                    2216.6      701.9   3.158  0.00245 **

R-sq.(adj) =    0.5   Deviance explained = 56.3%
GCV = 2.5744e+06  Scale est. = 2.2168e+06  n = 72
```

d. Meat content

```
Formula:
Tb ~ factor(SeedingDate) + factor(RW)

Parametric coefficients:
                               Estimate Std. Error t value Pr(>|t|)
(Intercept)                    0.043994   0.001795  24.507  < 2e-16 ***
factor(SeedingDate)2019-04-24 -0.001236   0.001216  -1.016   0.3141
factor(SeedingDate)2019-07-03 -0.002836   0.001217  -2.330   0.0237 *
factor(RW)3                    0.005294   0.002073   2.554   0.0136 *
factor(RW)5                    0.003275   0.002126   1.540   0.1296
factor(RW)7                    0.001053   0.002021   0.521   0.6045
factor(RW)9                   -0.004292   0.002065  -2.079   0.0426 *
factor(RW)11                   0.003848   0.002157   1.783   0.0803 .
factor(RW)13                   0.005252   0.002021   2.599   0.0122 *
factor(RW)15                   0.011430   0.002065   5.536 1.03e-06 ***

R-sq.(adj) =  0.562   Deviance explained = 62.7%
GCV = 1.7356e-05  Scale est. = 1.4557e-05  n = 62
```

e. Total biomass

```
Formula:
Tw ~ factor(SeedingDate) + factor(Vol)

Parametric coefficients:
                              Estimate Std. Error t value Pr(>|t|)
(Intercept)                    5.76472    0.79307   7.269 8.70e-10 ***
factor(SeedingDate)2019-04-24  4.31670    0.81607   5.290 1.81e-06 ***
factor(SeedingDate)2019-07-03 -0.09126    0.81607  -0.112 0.911336
factor(Vol)250                -3.29836    0.94623  -3.486 0.000924 ***
factor(Vol)450                -3.51878    0.94623  -3.719 0.000443 ***
factor(Vol)600                -4.78567    0.90220  -5.304 1.72e-06 ***

R-sq.(adj) =  0.495   Deviance explained = 53.3%
GCV = 8.0582  Scale est. = 7.3256    n = 66
```

Table S5. Scores of the PCA of Experiment 1 and Experiment 2. The more the value of the variable deviates from 0, the stronger the correlation with that principal component.

a. Experiment 1 (one common seeding date, three different sampling dates)

	PC1	PC2
cockle.density	0.1984594	-0.47849586
cockle.length	0.4438226	0.08402952
cockle.volume	0.4124381	-0.25762515
cockle.condition	0.1375601	0.45623348
cockle.biomass	0.2191078	-0.44994878
phytoplankton	0.2366963	0.45542436
microphytobenthos	-0.3790822	-0.15973812
median.grain.size	0.4014566	-0.12232774
siltfraction	-0.4070667	-0.19777625

b. Experiment 2 (three different seeding dates, one common sampling date)

	PC1	PC2
cockle.density	-0.3115103	0.19967980
cockle.length	-0.3730005	-0.25665329
cockle.volume	-0.3722640	0.06883643
cockle.condition	-0.2608368	-0.60574895
cockle.biomass	-0.3857685	-0.06545885
phytoplankton	-0.3020928	-0.37053833
microphytobenthos	0.3343078	-0.36314764
median.grain.size	-0.2349643	0.46208897
siltfraction	0.3875155	-0.18868195

C. Loadings of PCA (Figure S1)

a. Experiment 1 (one common seeding date, three different sampling dates).

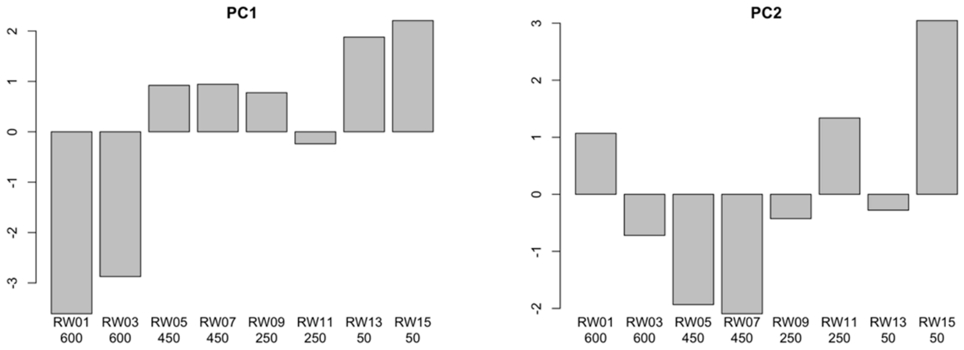

b. Experiment 2 (three different seeding dates, one common sampling date)

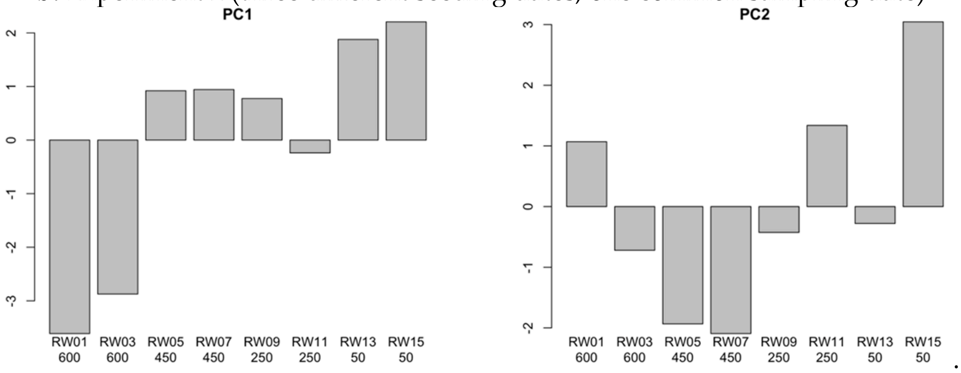

Figure S1. Loadings of 1st and 2nd axis of principal component analyses of the raceway coefficients on environmental conditions and cockle performance indices as derived from models M2 (see Table 1) on **(a)** Experiment 1 and **(b)** Experiment 2.
